# Prevalence of Type 2 Diabetes in the States of The Co-Operation Council for the Arab States of the Gulf: A Systematic Review

**DOI:** 10.1371/journal.pone.0040948

**Published:** 2012-08-08

**Authors:** Layla Alhyas, Ailsa McKay, Azeem Majeed

**Affiliations:** Department of Primary Care & Public Health, Imperial College London, London, United Kingdom; Universita Magna-Graecia di Catanzaro, Italy

## Abstract

**Aims:**

The recent and ongoing worldwide expansion in prevalence of Type 2 Diabetes (T2DM) is a considerable risk to individuals, health systems and economies. The increase in prevalence has been particularly marked in the states of the Co-operation Council for the Arab States of the Gulf (GCC), and these trends are set to continue. We aimed to systematically review the current prevalence of T2DM within these states, and also within particular sub-populations.

**Methods:**

We identified 27 published studies for review. Studies were identified by systematic database searches. Medline and Embase were searched using terms such as diabetes mellitus, non-insulin-dependent, hyperglycemia, prevalence, epidemiology and Gulf States. Our search also included scanning reference lists, contacting experts and hand-searching key journals. Studies were judged against pre-determined inclusion and exclusion criteria, and where suitable for inclusion, data extraction and quality assessment was achieved using a specifically-designed tool. All studies where prevalence of diabetes was investigated were eligible for inclusion. The inclusion criteria required that the study population be of a GCC country, but otherwise all ages, sexes and ethnicities were included, resident and migrant populations, urban and rural, of all socioeconomic and educational backgrounds. No limitations on publication type, publication status, study design or language of publication were imposed. However, we did not include secondary reports of data, such as review articles without novel data synthesis.

**Conclusions:**

The prevalence ofT2DM is an increasing problem for all GCC states. They may therefore benefit to a relatively high degree from co-ordinated implementation of broadly consistent management strategies. Further study of prevalence in children and in national versus expatriate populations would also be useful.

## Introduction

The World Economic Forum describes chronic diseases as one of the ‘top 6’ Global Risks [1] They carry enormous levels of morbidity and have become major causes of mortality. Diabetes mellitus is a chronic metabolic disorder caused by defects in insulin secretion, insulin action, or both. If ineffectively controlled, the resulting chronic hyperglycaemia is associated with numerous disabling complications. The International Diabetes Federation (IDF) 2010 estimate suggests that diabetes mellitus accounts for 6.8% of all-cause mortality in the 20–79 age group [2]. Over 90% of cases of diabetes mellitus are of type 2 diabetes mellitus (T2DM) [3], a form of diabetes characterised by insulin resistance with a relative or real insulin deficiency. Over the past 3–4 decades there has been a global expansion in the prevalence of T2DM, associated with population growth, ageing, urbanisation and lifestyle changes [4], [5]. These trends pose a particular risk to low- and middle- income countries, where most cases of diabetes and deaths from diabetes occur [5]; where a greater proportion of individuals affected by T2DM are of working age [6]; where changing demographics and lifestyles will lead to the greatest increases in prevalence; interventions are likely to be less widely available; and individuals generally pay a larger share of their health care costs.

The states of The Co-operation Council for the Arab States of the Gulf (GCC) have some of the highest rates of type 2 diabetes in the world. Five of the IDF's ‘top 10’ countries for diabetes prevalence in 2010 and in 2030 are in this region [1]. Currently, the IDF estimates suggest that in 2010 the ranking of countries by highest prevalence of diabetes starts as follows [2]:1. Nauru, 2. United Arab Emirates (UAE; prevalence 18.7%), 3. Kingdom of Saudi Arabia (KSA; prevalence 16.8%)), 4. Mauritius, 5. Bahrain (15.4%), 6. Reunion, 7. Kuwait (14.6%), 8. Oman (13.4%), 9. Tongo, 10. Malaysia.

Rates in Qatar are also relatively high (15.4% comparative prevalence). Prevalence estimates for 2030 (based only on anticipated changes in population size and demography [7]) suggest the same will be true then. The recent and rapid socio-economic development of the GCC countries has been associated with this rising prevalence. The IDF suggests that even in the absence of further economic development (that is, based on changes in population demography alone), the number of people with diabetes in its Middle East-North Africa region will increase 94% from 2010 to 2030. Only the Sub-Saharan African region is expected to see a greater increase in the number of cases of diabetes during this period [1].

Management strategies for T2DM are anticipated to be more effective when built around particular population and country parameters. We aim here to review the prevalence of T2DM in the GCC countries, to help establish that the problems in these states are broadly similar; and that their health systems are potentially suitable for implementation of similar management strategies. This is of particular current interest given the recent move within the GCC to co-ordinate control of diabetes care e.g. [8].

In addition to reviewing the general T2DM burden in these countries, we aimed to review, where possible, rates by age, sex, residential environment (urban/rural) and ethnicity. These were all anticipated – based on previous studies and preliminary scoping searches – as putative covariates of prevalence, and thus areas wherein sub-populations may benefit from specifically targeted management strategies.

## Methods

### Ethics statement

Ethical approval was not needed as the aim of this paper was to systematically review the literature on the prevalence of T2DM in the GCC countries.

### Review questions

A literature search was used to identify material relevant to the following review question:

What is the prevalence of T2DM in the GCC countries?

### Search

We developed a systematic review protocol (see appendix S1) using the Centre for Reviews and Dissemination guidelines [9]. Medline and Embase were searched separately on 15/07/2009 and the search was repeated on 03/07/2010 (via Dialog and Ovid, respectively; 1950 to July week 1 2010, and 1947 to July 2010) using terms identified from PICOS deconstruction of the above review questions such as diabetes mellitus, non-insulin-dependent, hyperglycemia, prevalence, epidemiology and Gulf States, and database- and manually-derived alternatives (appendix S2). The search strategy (see appendix S3) was trialled, reviewed by independent professional colleagues (E.H., K.P.), and updated before use. Further relevant studies were identified by searching the reference lists of the database-derived papers, contacting expert investigators, screening conference proceedings, citation searching and hand searching the International Journal of Diabetes and Metabolism and the Saudi Medical Journal, for the periods 1993–2009 and 2000–2010, respectively.

### Selection

The initial search produced 792 studies. After excluding duplicated studies (17 studies), the titles and abstracts were evaluated by one reviewer (L.A) to determine eligibility for full text screening. No limitations on publication type, publication status, study design or language of publication were imposed. However, we did not include secondary reports of prevalence, such as review articles without novel data synthesis. The inclusion criteria required that the study population be of a GCC country, but otherwise all ages, sexes and ethnicities were included, resident and migrant populations, urban and rural, of all socioeconomic and educational backgrounds. Studies of general-, working-, university- and healthcare attending- populations were included. We did not specify diagnostic criteria, but required that they would detect at least predominantly type 2 (rather than other forms of) diabetes, and they were incorporated into our data synthesis.

**Figure 1:**
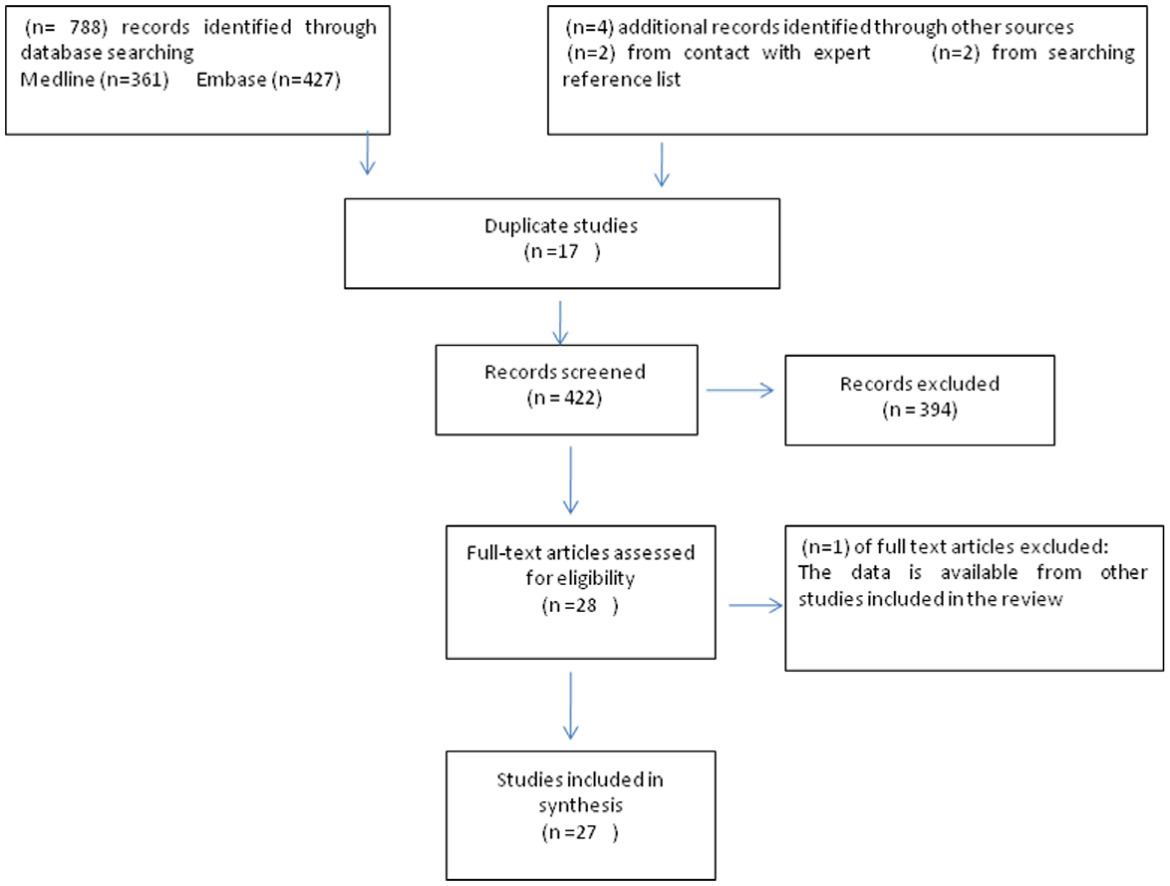
Flow chart of study selection process.

Twenty-eight studies were identified. The full texts of these studies were each examined by two reviewers (L.A and A.Mc). One study [10] was excluded as the data were already included in other studies [11]; [12], and no further (relevant) synthesis had been performed. The full text of a further study [13] could not be accessed, thus the abstract alone was used for review. Additionally, we could not fully access the data published in 3 studies [14]–[16], and the extracted data were therefore similarly limited. The selection process is summarised in Figure 1 and Figure S1.

### Data extraction/quality assessment

The data captured for each study included data relating to, (1) methods (study design, recruitment, measurement tools, analysis), (2) participant characteristics, (3) setting, and (4) outcomes (those observed, their definitions, results of analysis). Study quality was assessed using a checklist adapted from the Centre for Reviews and Dissemination guidelines [9]. Data extraction was performed, in duplication, by two reviewers (L.A and A.M) (see Table S1).

### Data synthesis

We were looking to estimate the prevalence of T2DM in the GCC countries between 1980 and 2009. To estimate the prevalence of T2DM, the related data was entered to STATA version 11 for statistical analysis. To assess the difference in prevalence of T2DM between different GCC countries and years, and to investigate the reasons for heterogeneity between the studies included in the review, a subgroup analysis was carried out. Subgroup analysis was performed for each country separately, and for years, which were classified as: (1) 1980–1989; (2) 1990–1999; and (3) 2000–2009. Publication year was used instead of the definite start year, as the last was not indicated in many studies. Furthermore, the measures of uncertainty (95% confidence intervals (CIs)) were calculated as meta-analysis was not appropriate to be conducted for the included studies in this systematic review due to the due to sampling, methodological and statistical variation of the studies [17].

Further data synthesis was designed around several proposals produced, for the most part, *a priori*, but also included an appraisal of potential association between diabetes and urban/rural residency, after preliminary scoping searches demonstrated that data pertaining to this were commonly reported.

These proposals were therefore:

1. prevalence of T2DM is increasing.
2. rates of T2DM in the GCC states are similar.
3. prevalence increases with advancing age.
4. there is a sex difference in prevalence.
5. there are differences in prevalence between urban and rural populations.
6. there are differences in prevalence between national and expatriate populations.

In addition, prevalence in children was separately considered.

Association of diabetes with BMI and relationship to income/socioeconomic status were not examined in this review.

In consideration of the above proposals, synthesis included summarising the results of the data extraction process, considering the strength of evidence relating to each of our questions, and examination of results inconsistent with our formed suggestions (Figure S2).

## Results

Twenty-seven journal-published studies were identified for inclusion. A summary is provided in Table 1, and further details are available in Tables S2 (A) and (B). The studies were carried out (where reported) and published between 1982 and 2009. Six studies were published and undertaken in the 1980s, 13 in the 1990s, 8 in the 2000s. Eleven studies were of Saudi populations, 3 Kuwaiti, 2 Bahraini, 6 Emirati, 4 Omani and 1 Qatari. Sample sizes ranged from 336 to 600132. All were cross-sectional studies. In 17 cases, the general population was the target population; in 4 cases, the sample was patients registered with primary health care centres. Three studies estimated prevalence in working populations with or without dependants, one in a university population, one a population of schoolchildren, and one a ‘clinic-attending population’ (clinic type unclear). In one working population, and the university population, the sample was entirely male.

**Table 1:** Summary of included studies.

Ref/dates of study*	Country	Sample size	Prevalence rate	Age & prevalence	Age & sex
Bacchus et al/NR (1982)	KSA	1385	No diabetic cases in people <24 years 0.3%: age group 25–34 years 2.6%: age group 35–44 years 9.6%: age group 45–54 years 1%: age group 55–64		
Anokute et al/1985–1987	KSA	3158	Overall prevalence ‘positive’ FBG (unconfirmed DM): 6.0%	The age specific prevalence increased with age to a maximum of 33.8% for the age group ≥50 years.	
Fatani et al/NR (1987)	KSA	5222	overall prevalence DM 4.3%		prevalence DM lower in men (2.9%) vs. women (5.9%; p<0.001)
Balasy & Radwan/1989^2^	UAE	1517	Age adjusted prevalence rate for DM: 5.69% Prevalence of DM among males vs. females: 1.81% vs. 2.58% respectively	The age specific prevalence of DM was steadily increasing until age 59 in both genders	Prevalence of DM among males vs. females: 1.81% vs. 2.58% respectively
Abu-Zeid and Al-Kassab/1989	KSA	1419	Overall prevalence DM 4.6%		Prevalence of DM in men (5.5%) than women (3.6%; p<0.05); overall prevalence IGT: 3.7%; higher in women (4.9%) vs. men (2.5%; p<0.01)
Abdella et al/1989–1990	Kuwait	261387	Overall prevalence DM: 7.6%	prevalence generally increased with age in both sexes in both areas (rural and urban) (no test for significance)	Prevalence was generally greater in females (no test for significance)
El-Hazmi et al/1991	KSA	23493	The prevalence of T2DM: 4.9% The prevalence of IGT: 0.7%	The prevalence of DM peaked in the age group≥30 years (P<0.001)	
El-Hazmi et al/1991	KSA	2060	The overall prevalence of T2DM: 6.89%; IGT: 0.77%		
Al-Lawati & Mohammed/1991	Oman	4682	Prevalence of DM: 10.5% by WHO criteria, 8.2% by ADA criteria Prevalence of IGT 10.5% by WHO criteria, 5.7% by ADA criteria		
Al-Nuaim/1991–1993	KSA	13177	Overall prevalence DM: 12% in urban males, 7% rural males, 14% urban females, 8% rural females Overall prevalence IGT: 10% in urban males, 8% in rural males, 11% in urban females, 8% in rural females		
Mahfouz et al/1993	KSA	600132	Prevalence DM 9.7% in males, 9.8% in females Prevalence IGT 8.1% in males, 12.9% in females		Prevalence DM 9.7% in males, 9.8% in females
Al-Shammari et al/1993–1994	KSA	2990	Overall prevalence DM 12.2%		
Glasgow et al/1995	UAE	<33% of > 29809	The rate of DM from the two databases for UAE citizens >30 years: 5.7% and 11.2%	In one of the databases the rate of DM increased from 1.4% in the age group 30–34 years to between 8.9% and 11% in the age group >40–44 years.	
Al-Mahroos & McKelcue/1995–1996	Bahrain	2002	Overall prevalence DM: 29.8%		prevalence DM in males 40 – 49: 22.9%; 50 – 59: 29.6%; in females 50 – 59: 35.4%; 60 – 69: 37.6%
Townsend/NR (1997)	UAE	336	Overall prevalence unclear 6.2%, >30 years found to be diabetic 19% of subjects >20 years had IGT in previously undiagnosed: 4.8		
El-Hazmi et al/NR (1998)	KSA	25337	The prevalence of T2DM and IGT: 5.63% and 0.5% respectively in males, in females: 4.53% and 0.72% respectively	The prevalence of T2DM was 0.12% and 0.79% in people<14 and people aged 14–29 years respectively. In the age ≥60, the rate increased to 28.8% and 24.9% in males and females respectively.	Prevalence of T2DM in males vs. Females respectively: 5.63% vs. 4.53%
Al-Nozha et al/1995–2000	KSA	16197	Overall prevalence DM 23.7%		prevalence higher in males: 26.2% (95% CI 25.2 – 27.2) vs. females 21.5% (95% CI 20.6 – 22.4; p<0.0001) (significance unclear); overall prevalence IFG 14.1% (no gender difference)
Malik et al/1999–2000	UAE	5844	overall prevalence DM: 21.4% (95% CI 20.4 – 22.4%) Prevalence in UAE citizens 25%, expats 13 – 19%		prevalence in men 20.4% (18.8 – 22.0%); prevalence in women 22.3% (20.9 – 23.7%)
Asfour et al/2000	Oman	5096	Crude prevalence of DM: 10% in both gender.	In both gender, the prevalence of IGT increased with age, it peaked in the age group (60–69)	(11) Asfour et al/2000
Al-Asi/2000	Kuwait	3282	Overall prevalence of DM: 17%		
Al-Mahroos and Al-Roomi/NR (2001)	Bahrain	2013	overall prevalence DM 30%		
Moussa et al/2000–2002	Kuwait	128918	Overall prevalence DM: 34.9 per 100000 (95% CI 24.7 – 45.1)	Significantly higher prevalence T2DM with advancing age (p = 0.026).	Prevalence of DM in males 47.3 per 100000 (CI 28.7–65.8); females 26.3 per 100000 (CI 14.8–37.8). significantly higher prevalence T2DM in males (p = 0.05)
Al-Lawati et al/NR (2002)	Oman	5838	Prevalence of DM among male and female: 11.8% vs. 11.3% respectively (P = 0.275)	Prevalence of DM rose with age and exceeded 20% in both genders at the age of 50 years	IGT was more prevalence among males than females 7.1% vs. 5.1% (P<0.001)
Baynouna et al/2004–2005	UAE	817	Overall prevalence DM 23.3%; prevalence by age and gender: males: 5.1% 20 – 29 years, 11.1% 30 – 39 years, 29.5% 40 – 49 years, 35.5% 50 – 59 years, 55.9% >60 years; females: 1.7% 20 – 29 years, 5.3% 30 – 39 years, 26.2% 40 – 49 years, 27.1% 50 – 59 years, 43.3% >60 years		
Saadi et al/2005–2006	UAE	2396	Overall prevalence DM: 10.2% (9.4% in males, 11.1% in females); prevalence in 30 – 64 years population: 20.6% (17.7% in males, 22.1% in women)		
Al-Moosa et al/NR (2006)	Oman	5840	overall prevalence DM: 11.6% (11.8% in males, 11.3% in females		
Bener et al/2009	Qatar	1117	Overall prevalence DM: 16.7% (15.2% males, 18.1% females)	Age significantly associated with DM (p = 0.0001, multiple logistic regression analysis); peak age DM 40 – 49 years (58%)	

NR: not reported.

(*): the publication year was used instead when the study date was not reported.

### Prevalence of T2DM and association with time and countries

21 studies were included in the sub-analysis based on methodological consideration [16], [18]–[38]]. The sub-analysis suggested that estimated prevalence had increased across the three time periods listed in the data analysis section respectively (3.58% [95% CIs, 1.94–5.23; 2 studies]17, 18; vs. 4.01% [95% CIs, 3.58–4.43; 10 studies] 11,16,19,20,21,24–28; vs. 5.06% [95% CIs, 4.02–6.09%;10 studies ] 12, 22, 29, 30–33, 35, 36, 37). The differences in the estimated prevalence rate of T2DM in the GCC countries between the three periods was not statistically significant p = 0.9.

Subgroup analysis by country indicated that the estimated prevalence rates of T2DM between GCC countries are comparable. The lowest estimated prevalence rate was found in KSA 4.01% [95% CIs, 3.60–4.43; 10 studies] 16, 18–21, 25–28, 31; followed by Oman 4.5% [95% CIs, 3.16–5.85; 4 studies] 33, 11, 12, 22. Bahrain, in contrast, had the highest estimated prevalence rate of T2DM among GCC countries at 5.17% [95% CIs, 2.48–8.93; 2 studies] 28, 29. However, the estimated prevalence rates between Qatar, UAE and Kuwait were close (5.12 [95% CIs, 0.39–9.85; 1 study] 38; vs. 5.10% [95% CIs, 2.90–7.30; 3 studies] 35, 36, 31; vs. 5.14% [95% CIs, 1.45–8.82; 1 study] 32; respectively).

### Prevalence of DM and age

Four studies (all studies in which testing was well described) demonstrated a significant association between advancing age and prevalence of diabetes [25], [36]–[38]. There was otherwise, where reported, an apparently similar association of unclear significance, or in some cases, such an association until 40 – 49 [38], 59 [13] or 60 [15] years, after which point the prevalence appeared to decrease, or fluctuate. Fatani *et al* [19], report an association (multiple logistic regression analysis; P<0.0001) between age and blood glucose levels.

### Prevalence of DM and sex

Significant sex differences were reported in 6 studies (including that of schoolchildren). All except one relatively old report [19] were in favour of a male predominance [21], [24], [27], [31], [35]. In 9 further studies, however, higher prevalence, of undetermined significance (or close to significance: [29]), was observed in females. This was the case for males in 2 studies. A further 3 studies showed no significant gender difference.

### Prevalence of DM and residential environs

Urban versus rural prevalence was commented on in 5 studies [27], [22], [31], [34], [21]. All except the oldest study [21] demonstrated higher prevalence in urban.

### Prevalence of DM in children

Prevalence in children was consistently reported to be low: 0.035% [35], 0.027% [24], 0.033% and 0.099% (in urban and rural populations, respectively; [22]).

### Prevalence of DM in national/expatriate populations

The prevalence of diabetes in UAE-resident expatriate populations, versus that in UAE citizens, was considered in only one study [32]. The UAE citizens appeared to have relatively high rates of disease, although no statistical methods were employed to test this suggestion.

## Discussion

We reviewed the prevalence of T2DM in the GCC region, and any differences by country, age, sex, urban-rural residence and ethnicity. We identified 27 papers for review, and descriptive results from the review indicated that prevalence of T2DM in the GCC countries ranged between 4.3%–34.9% for studies published between 1980 and 2009. The estimated prevalences of T2DM in Qatar, UAE and Kuwait were close as the included studies were carried out in the same period between 2000 and 2008; however lower prevalences were observed in KSA and Oman as six of the studies included were carried out between 1990 and 1999, two studies in 1980s and two studies in 2000. The higher rates seen in Bahrain; however could be a result of the documented high prevalence rates in the two studies included in the sub-analysis by country (29.8% and 30%).

Our study was also suggestive that prevalence increases with age (at least to 50–60 years), and that urban residence is associated with higher prevalence. The importance of age as a risk factor is consistent with previous data, from many contexts [39], [40].

The observed high prevalence of diabetes in the GCC states is likely to be associated with the high prevalences of risk factors for T2DM in this region. The International Diabetes Federation suggests age, obesity, family history, physical inactivity, race and ethnicity, and gestational diabetes to be risk factors for T2DM [4]. We recently observed that overweight, obesity and hyperglycaemia are present at high levels in the GCC states [41]. We also noted the aging of the GCC populations, which is a likely contributory factor to the increasing prevalence.

### Quality consideration

There were some inconsistencies in our tabulated results: both generally, and within the country of investigation. The studies of El-Hazmi *et al* [16] and Mahfouz *et al* [23] produced relatively low results, inconsistent with the general trend. The El-Hazmi *et al* [16] sample is 39.1% children <14 years, which may account for the low rates. The authors report a ‘significant’ increase in prevalence with age, but we could not access the full data and the statistical methods used were not well-described. We have suggested a higher prevalence with advancing age of unclear significance, but with rates of 0.12% and 0.79% in those <14 and 14 – 29 years, respectively, and rates of 28.82% (males) and 24.92% (females) in those >60 years, this is potentially rather conservative. We believe that this study is consistent with the others listed, but it does highlight that a number of other prevalences we have tabulated are also relatively low because they include children [19], [20], [21]. Indeed, the prevalences in these populations are much higher when children are removed from the calculation, although insufficiently to interrupt the general trends observed. This cannot however, explain the low rates reported by Mahfouz *et al* [*23*], and so we consider that these may be due to inclusion of only previously diagnosed people with diabetes (and omission of the often substantial ‘undiagnosed’ population), but concede that the result could still be relatively low, and of importance given the sample size.

We cannot extend our observed association between type 2 diabetes and age to children. Low prevalences of T2DM in children have been reported since the late 1990s (date of first identified study). However, the data are few; insufficient to evaluate the possibility that prevalence in children is increasing, as has been observed in other countries [42], [43].

The relationship between T2DM and sex was unclear. We noted, where tested, predominance in males. Wild *et al* [7] have reported this to be the case (‘globally’) for individuals <60 years. Even where these differences may exist, however, they generally appear to be slight. By contrast, we did observe higher prevalence associated with urban (cf. rural) residence, which again has been observed by others e.g. [44], [45].

Only a few of our included studies excluded patients with type 1 diabetes (including the study in schoolchildren) and/or pregnant women. It is therefore likely that in the majority of samples tested, the prevalence suggested includes small numbers of type 1, gestational, and potentially other forms of diabetes.

The majority of studies relied at least in part on the various World Health Organization (WHO) criteria for diagnosis. There is mild variation in definition by edition of WHO criteria, with discrepancies producing differences in estimations of the extent demonstrated by Al-Lawati and Mohammed [24], and only the later editions (1998 onwards) are consistent with those of the American Diabetes Association. Some studies, however, used definitions of diabetes and methodological approaches that led to results relatively difficult to use comparatively. Some relied on previous records alone to make diagnoses (where the diagnostic criteria used were often unclear), and so potentially missed an ‘undiagnosed’ section of the diabetes population, which has been reported to be potentially substantial [12], [14], [30], [33], [38], [39]. By contrast, relying purely on blood sampling to estimate of prevalence may have missed a significant number of cases of treated, well-controlled disease [21]. In addition, one study [37] omitted patients with secondary dyslipidaemias and may therefore have underestimated prevalence; another [15] measured prevalence in primary care consultations rather than prevalence within the population. There were also concerns that loss of difficult cases to secondary care [29], or identification only of cases sufficiently severe to merit secondary care [23], may have resulted in estimated prevalences providing relatively poor estimates for the general population.

### Limitations of review

The heterogeneity of the reviewed studies, and variable availability of sub-group data, was a major limitation in our review process. All of our reviewed studies were published in English. Clarity of reporting was a relatively frequent quality issue, but we did not exclude any studies on this basis. Indeed, with such paucity of data and inability to draw more than broad conclusions anyway, we included even studies without full data availability, and one where only the abstract could be accessed.

Although five studies [26], [29], [30], [36], [38] had high rate of loss of follow-up (>20%), they were included in the review. In four of these studies [26], [29], [30], [34], the target number of subjects that were supposed to take part in these studies was unreachable. For instance, in two studies (30 and 36) 382 and 861 subjects respectively were not resident at the address given. Other reasons for high loss to follow-up were participants' death, travelling abroad, refusal to participate in the studies, and exclusion based on health grounds. Bener et al. [38] was included although details on the high rate of loss of follow up was not mentioned because it is the single study carried out in Qatar, and excluding it from the review would not help us draw an estimated prevalence rate of T2DM in this country. All of these factors impact on the strength and confidence of our proposals.

### Implications

The relatively high levels of T2DM in the GCC region, and increasing prevalence, suggest that novel, or more widespread, management strategies will be important to averting an increasingly unmanageable problem. This may be particularly so given the observed associations with urban residence and age, within a context of continued urbanisation and unfavourable trends in population demography. The nature of the problem is probably similar across the different GCC states (with the possible exception of Oman, for which data are limited). Potentially, then, cross-implementation of management strategies would provide relatively high levels of success, and a co-ordination of effort would likely be relatively cost-effective. Cost is particularly important given the size of the problem, the observed impact on the working population, and the nature of migrant populations within the GCC region.

The migrant populations contribute greatly to the currently high rates of population growth in these countries. General prevalence could thus be hugely influenced by differential disease rates between national and expatriate populations. This is particularly important to estimates of future rates of disease as these are usually based – at least in part – on predicted changes in population demographics. As the GCC countries have strict naturalisation policies, and the vast majority of expatriate workers are not national citizens, these countries are at relatively high risk of fluctuation in population size and structure, and predictions regarding demography are thus relatively difficult to make. Economic change could have a particularly strong impact on population structure, and building such possibilities into strategies for disease management, when this is itself of significant economic status, is important.

Given these issues, we find the observed infrequent consideration of ethnicity as a variable particularly striking, and anticipate that continued study of this issue would be useful. Study of physical inactivity – another risk factor for T2DM – may also be useful. Finally, we expect that study of prevalence in children would be helpful, particularly given the recent rise in childhood prevalence reported elsewhere, as the available data are minimal. Longitudinal studies in both children and adults are desirable, as longitudinal data are lacking and such studies would be the optimal way to observe changes in prevalence with time.

## Conclusions

This is the first systematic review has been undertaken in the countries of the GCC to estimate the prevalence rate of T2DM. There were several methodological challenges; in particular, the different populations studied and methods used to assess glycaemic status. This review presents the high prevalence of T2DM in the region and the increasing burden of this disorder over time in the GCC countries, which is in line with statistics from the IDF on the “top 10” countries for diabetes prevalence in 2010 and in 2030. Primary prevention strategies may be useful in reducing its incidence in the GCC region. Finally, we recommend further epidemiological studies to estimate the prevalence of T2DM in the area and to observe any changes in prevalence rate over time, using longitudinal data collection in higher-quality studies that would give accurate statistics on diabetes prevalence, including prevalence in key population sub-groups.

## Supporting Information

Figure S1 **PRISMA 2009 Flow Diagram.**
(DOC)

Figure S2 **PRISMA 2009 checklist.**
(DOC)

Table S1 **Study quality assessment.**
(DOCX)

Table S2 (A) Summary of prevalence of type 2 diabetes in the GCC region. (B) Summary of prevalence of type 2 diabetes in the GCC region.
(DOCX)

Appendix S1 **Search Protocol.**
(DOCX)

Appendix S2 **Research questions using PICO.**
(DOCX)

Appendix S3 **Search strategy.**
(DOCX)
