# Supplementary material for: Prevalence of Type 2 Diabetes in the States of The Co-Operation Council for the Arab States of the Gulf: A Systematic Review
Source: PLoS One. 2012 Aug 8;7(8):e40948. doi: 10.1371/journal.pone.0040948 (PMC3414510; doi:10.1371/journal.pone.0040948)
Supplement: Figure S1 — PRISMA 2009 Flow Diagram. (DOC) [file pone.0040948.s001.doc]

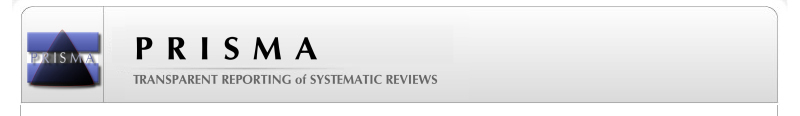
**Figure S1: FPRISMA 2009 Flow Diagram**

**Screening**

**Included**

**Eligibility**

**Identification**

Records identified through database searching
(n =788 )

Additional records identified through other sources
(n = 4 )

Records after duplicates removed
(n = 775 )

Records screened
(n = 775 )

Records excluded
(n = 394 )

Full-text articles assessed for eligibility
(n = 28 )

Full-text articles excluded, with reasons
(n = 1 )

Studies included in qualitative synthesis
(n = 27 )
