# Supplementary material for: Prevalence of Type 2 Diabetes in the States of The Co-Operation Council for the Arab States of the Gulf: A Systematic Review
Source: PLoS One. 2012 Aug 8;7(8):e40948. doi: 10.1371/journal.pone.0040948 (PMC3414510; doi:10.1371/journal.pone.0040948)
Supplement: Table S1 — Study quality assessment. (DOCX) [file pone.0040948.s003.docx]

| **Ref/dates of study** | **Quality assessment checklist (1)** |
| --- | --- |
| Al-Lawati &Jouilahti/ 1991 | 1-Y, 2-Y, 3-Y, 4-Y, 65-N, 6-N, 7-NA |
| Asfour et al/ 2000 | 1-Y, 2-Y, 3-Y, 4-Y, 5-N, 6-N, 7-NA |
| Al-Lawati et al/ NR  **Table S1: Study quality assessment** | 1-Y, 2-Y, 3-Y, 4-Y, 5-N, 6-N, 7-NA |
| Balasy & Radwan/ 1989^2; 9^ | Full article not available for assessment |
| Townsend/ NR | 1-Y, 2-Y, 3-Y, 4-N, 5-Unclear, 6-N, 7-NA |
| Glasgow et al/ 1995 | 1-N, 2-partially, 3-N, 4-N, 5-N, 6-Y, 7-NA |
| El-Hazmi et al/ NR | 1-Y, 2- Incomplete, 3-Y, 4-Y, 5-Y, 6-N, 7-NA |
| Bacchus et al/ NR | 1-Y, 2-Incomplete, 3- Y, 4- Y, 5-N, 6- Some, 7-NA |
| Fatani et al/ NR | 1-Y, 2-Y, 3-Y, 4-Y, 5-N, 6-N, 7-NA |
| Anokute et al/ 1985-1987 | 1-Y, 2-Y, 3-Y, 4-Y, 5-Y, 6-N, 7-NA |
| Abu-Zeid and Al-Kassab/ 1989 | 1-Y, 2-Incomplete, 3-Y, 4-Y, 5-N, 6-N, 7-NA |
| Abdella et al/1989-1990 | 1-Y, 2-Y, 3-Y, 4-N, 5-Y, 6-N, 7-NA |
| Al-Lawati & Mohammed / 1991 | 1-Y, 2-Y, 3-Y, 4-Y, 5-N, 6-N, 7-NA |
| Mahfouz et al/1993 | 1-Y, 2-Y, 3-Y, 4-Incomplete, 5-N, 6-N, 7-NA |
| El-Hazmi et al/ 1991 | 1-Y, 2-Y, 3-Y, 4-Y, 5-N, 6-N, 7-NA |
| El-Hazmi et al/1991 | 1-Y, 2-Y, 3-Y, 4-Y, 5-N, 6-Some, 7-NA |
| Al-Nuaim/ 1991-1993 | 1-Y, 2-Incomplete, 3-Y, 4-Y, 5-Y, 6-Some, 7-NA |
| Al-Shammari et al/ 1993-1994 | 1-Y, 2-Y, 3-Y, 4-Y, 5-Y, 6-N, 7-NA |
| Al-Mahroos & McKelcue/ 1995-1996 | 1-Y, 2-Y, 3-Y, 4-Y, 5-N, 6-N, 7-NA |
| Al-Mahroos and Al-Roomi/ NR | 1-Y, 2-Y, 3-Y, 4-Y, 5-Y, 6-N, 7-NA |
| Al-Nozha et al/ 1995-2000 | 1-Y, 2-Incomplete, 3-Y, 4-N, 5-Y, 6-N, 7-NA |
| Malik et al/ 1999-2000 | 1-Y, 2-Y, 3-Y, 4-Y, 5-Y, 6-N, 7-NA |
| Al-Asi/ 2000 | 1-Y, 2-Y, 3-Y, 4-Y, 5-N, 6-N, 7-NA |
| Al-Moosa et al/ NR | 1-Y, 2-Incomplete, 3-Y, 4-Y, 5-N, 6-Y, 7-NA |
| Moussa et al/ 2000-2002 | 1-Y, 2-Incomplete, 3-Y, 4-Y, 5-N, 6-Y, 7-NA |
| Baynouna et al/ 2004-2005 | 1-Y, 2-Y, 3-Y, 4-N, 5-Y, 6- N, 7-NA |
| Saadi et al/ 2005-2006 | 1-Y, 2-Y, 3-Y, 4-Y, 5-N, 6-Y, 7-NA |
| Bener et al/ 2009 | 1-Y, 2-Y, 3-Y, 4-Y, 5-Y, 6-Some, 7-NA |

Table S1: Quality assessment

R: rural residents; U: urban residents; SR: self-reported diagnosis; PD: previous diagnosis; CBG: capillary blood glucose; RBG: random blood glucose

Quality assessment checklist (1)

1. ^Was the aim of the study stated clearly?^
2. ^Was the methodology stated? And was it appropriate?^
3. ^Were appropriate methods used for data collection and analysis?^
4. ^Was the data analysis sufficiently rigours?^
5. ^Were preventive steps taken to minimize bias?^
6. ^Were limitations of the study discussed?^
7. ^In systematic review, was search strategy adequate and appropriate?^
