# Supplementary material for: Prevalence of Type 2 Diabetes in the States of The Co-Operation Council for the Arab States of the Gulf: A Systematic Review
Source: PLoS One. 2012 Aug 8;7(8):e40948. doi: 10.1371/journal.pone.0040948 (PMC3414510; doi:10.1371/journal.pone.0040948)
Supplement: Table S2 — (A) Summary of prevalence of type 2 diabetes in the GCC region. (B) Summary of prevalence of type 2 diabetes in the GCC region. (DOCX) [file pone.0040948.s004.docx]

| **Ref/dates of study** | **Country** | **Sampling** | | | **Population characteristics** | | |
| --- | --- | --- | --- | --- | --- | --- | --- |
|  |  |  |  |  | **% male** | **Age Range (years)** | **Nationality; U/R** |
|  |  | **Population sampled** | **Response rate** | **Sample size** |  |  |  |
| Al-Lawati &Jouilahti/ 1991 | Oman | Households | 91% | 5096 | 41.9 | 20 to > 80 | U/R mix |
| Asfour et al/ 2000 | Oman | Households | 93% (males 92%, females 94%) | 5838 | 49.8 | 20 to > 80 | Omani; U/R mix |
| Al-Lawati et al/ NR | Oman | Households | 83% | 5847 | Urban:  48.8; Rural: 50.3 | 20 to > 60 |  |
| Balasy & Radwan/ 1989^2; 9^ | UAE | Health centre^4^ |  | 15175 | NR^8^ |  |  |
| Townsend^2; 9^/ / NR | UAE | PC |  | 336 | NR ^8^ | >20 | U/R mix |
| Glasgow et al^2^ / 1995 | UAE | PC |  | < 33 % of > 29809 | NR^8^ | >30 | U/R mix |
| El-Hazmi et al^2^ / NR | KSA | Households | 95% | 25337 | 46.2 | < 14 to > 60 | Saudi |
| Bacchus et al/ NR | KSA | Working ^3^ |  | 1385 | 100 | < 15 to > 65 | Saudi, R |
| Fatani et al/ NR | KSA | Households | - | 5222 | 53.1 | 15 to >55 |  |
| Anokute et al/ 1985-1987 | KSA | University | - | 3158 | 100 | Mean ages by subgroup: 31, 23 and 41 years | 86.3 % Saudi; U |
| Abu-Zeid and Al-Kassab/ 1989 | KSA | Households | 87% | 1419 | 49.4 | 10 to > 60 | 98 % Saudi; ‘semiurban’/R |
| Abdella et al/1989-1990 | Kuwait | Households | - | 261387^7^ | Approximately: 50 | < 20 to > 60 | Kuwaiti; U/R mix |
| Al-Lawati & Mohammed / 1991 | Oman | Households | - | 4682 | 42.8 | >20 | U/R mix |
| Mahfouz et al/1993 | KSA | PC | - | 600132 | NR^8^ | > 5 |  |
| El-Hazmi et al/ 1991 | KSA | Household | - | 23493 | 46 | 2-70 | Saudi |
| El-Hazmi et al/1991 | KSA | Household | Roughly 95% | 2060 | 48.5 | 14 to >50 | Saudi |
| Al-Nuaim/ 1991-1993 | KSA | Households | 69% | 13177 | 52 | 15 to >60 | Saudi |
| Al-Shammari et al/ 1993-1994 | KSA | Working^5^ | - | 2990 | NR^8^ | Unclear | 94.7% Saudi |
| Al-Mahroos and McKelcue/ 1995-1996 | Bahrain | Households | 59-70% | 2002 | 58.6 | Males: 40 – 59  Females: 50 – 69 | Bahraini |
| Al-Mahroos and Al-Roomi/ NR | Bahrain | Households | 59-70% | 2013 | 58 | Males: 40 – 59  Females: 50 – 69 | Bahraini |
| Al-Nozha et al/ 1995-2000 | KSA | Households | 98.2% | 16197 | 47.6 | 30-70 | Saudi |
| Malik et al/ 1999-2000 | UAE | Households | 89% | 5844 | 42.7 | < 14 to > 60 | UAE residents; '80 % U' |
| Al-Asi/ 2000 | Kuwait | Working^6^ | 89.4% | 3282 | 85 | 54 % < 40 | 62 % Kuwaiti |
| Al-Moosa et al/ NR | Oman | Working | 96% | 5847 | Urban:  48.8; Rural: 50.3 | 20 to > 60 |  |
| Moussa et al/ 2000-2002 | Kuwait | School-children |  | 128918 | 41 | 6 – 18 | Kuwaiti |
| Baynouna et al/ 2004-2005 | UAE | Households | 40.8% | 817 | 49.3 | 20 to > 60 | Emirati |
| Saadi et al/ 2005-2006 | UAE | Households |  | 2396 | 49.1 | 18 to > 70 | Emirati; U |
| Bener et al/ 2009 | Qatar | PC | 77.9% | 1117 | 51.1 | 20 - 59 | U/  ‘semiurban’ |

**Table S2 (A): Summary of prevalence of type 2 diabetes in the GCC region**

Summary of cross-sectional studies investigating prevalence of type 2 diabetes within populations of the GCC region

R: rural residents; U: urban residents; SR: self-reported diagnosis; PD: previous diagnosis; CBG: capillary blood glucose; RBG: random blood glucose

1. Abstract only reviewed (full paper not available); 2.Full data unavailable; 3. Government/ municipal salaried workers; 4. Nature of clinic unclear; 5. Employees of Saudi National Guard and dependents; 6. Employees of Kuwait Oil Company; 7. N = 130364 (urban group), 131023 (rural group); 8. Males and females included; 9. Pilot study; 10. Rates of diabetes as subject of consultation (not rates of diagnosis) investigated; 11. Until 59 years, no trend thereafter; 12. Falling after 60 years in one clinic; 13. Age significantly associated with DM (multiple logistic regression analysis): peak age DM 40 - 49 years

| **Ref/dates of study** | **Diagnostic criteria** | | **Results** | | **Study limitations** |
| --- | --- | --- | --- | --- | --- |
|  | **Criteria followed** | **Method of screening** | **Prevalence of DM** | **Others** |  |
| Al-Lawati &Jouilahti/ 1991 | WHO 1985 |  | Overall prevalence of DM ( Males: 9.7  Females: 9.8) | Mean BMI increased from 24.3kg/m2 in 1991 to 25.2/kg/m2 in 2000 among males (P<0.001). Among females, it decreased from 26.3 kg/m2 to 25.8 kg/m2 (P<0.001)  In both gender, mean BMI increased with age and peaked in the age group (40-49) | No statistical analysis; Characteristics of population studied unclear |
| Asfour et al/ 2000 | WHO 1999/ SR | OGTT | Crude prevalence of DM: 10% in both gender.  Prevalence of DM rose through life in both gender to a maximum of > 30% | IGT was more common in female than male: 13% vs. 8% respectively.  In both gender, the prevalence of IGT increased with age, it peaked in the age group (60-69) | Limitation of the study not discussed  Steps taken to minimize bias not discussed |
| Al-Lawati et al/ NR | WHO 1999 | FPG | Prevalence of DM among male and female: 11.8% vs. 11.3% respectively (P=0.275) | Prevalence of DM rose with age and exceeded 20% in both genders at the age of 50 years  IGT was more prevalence among males than females 7.1% vs. 5.1% (P<0.001) | Dates of investigation unclear |
| Balasy & Radwan/ 1989 | NR |  | Age adjusted prevalence rate for DM: 5.69%  Prevalence of DM among males vs. females: 1.81% vs. 2.58% respectively | The age specific prevalence of DM was steadily increasing until age 59 in both genders. |  |
| Townsend/ NR | WHO 1980 | Random capillary blood sampling in non-diabetic subjects was compared with OGTT in known diabetic samples | Overall prevalence unclear  6.2% , > 30 years found to be diabetic  19% of subjects >20 years had IGT  - in previously undiagnosed: 4.8 | There was no apparent correlation of undiagnosed DM with BMI | Dates of investigation unclear; Characteristics of study population not well documented (e.g. sex) |
| Glasgow et al/ 1995 | Previously diagnosed | NR | The rate of DM from the two databases for UAE citizens >30 years: 5.7% and 11.2% | In one of the databases the rate of DM increased from 1.4% in the age group 30-34 years to between 8.9% and 11% in the age group > 40-44 years.  At the other database, the DM rates increased from 21% in the age group 30-34% to 21.6% in the age group 60-64, and decreased to 4.2% in people ≥ 65 years. | Rates of diabetes as subject of consultation (rather than rates of diagnosis) investigated.  Diagnosis of DM not confirmed |
| El-Hazmi et al/ NR | WHO 1980/1985 | FPG | The prevalence of T2DM and IGT: 5.63% and 0.5% respectively in males, in females: 4.53% and 0.72% respectively | The prevalence of T2DM was 0.12% and 0.79% in people< 14 and people aged 14-29 years respectively. In the age ≥ 60, the rate increased to 28.8% and 24.9% in males and females respectively. | Dates of investigation unclear; Results by sex (*table 3) not available |
| Bacchus et al/ NR | WHO 1980 | FPG and OGGT | No diabetic cases in people <24 years  0.3%: age group 25-34 years  2.6%: age group 35-44 years  9.6%: age group 45-54 years  11%: age group 55-64 | 65% of people with DM were overweight vs. 26% of people without DM | Sample not representative for the whole population  Recruitment process not specified |
| Fatani et al/ NR | Random CBG > 11 mM/ WHO 1980 (OGTT) | ICT, OGGT | overall prevalence DM 4.3 %; prevalence DM lower in men (2.9 %) vs. women (5.9 %; p< 0.001)  Overall prevalence IGT 1.1 %; | In subjects > 15 years, prevalence 4.0 % in men, 9.5 % in women (p < 0.001)  prevalence of DM higher in higher income groups (p < 0.001)  Age, income and BMI were associated with blood glucose by multiple logistic regression analysis (p < 0.004, p < 0.0001 and p < 0.045 respectively) | Dates of study unclear  Selection method of houses for sampling not reported |
| Anokute et al/ 1985-1987 | 2x fasting CBG > 7.8 mM | FPG | overall prevalence 'positive' FBG (unconfirmed DM): 6.0 % | The age specific prevalence increased with age to a maximum of 33.8% for the age group ≥ 50 years. | Recruitment procedure not reported  No statistical analysis  Sample not representative for the whole population  (male university community) |
| Abu-Zeid and Al-Kassab/ 1989 | PD/ 2-hour fasting post-meal CBG > 11.1 mM | FPG and IGT | Overall prevalence DM 4.6 %, higher in men (5.5 %) than women (3.6 %; p < 0.05);; overall prevalence IGT: 3.7 %; higher in women (4.9 %) vs. men (2.5 %; p < 0.01) | Prevalence by sex was statistically significant (P<0.05)  Prevalence rose with age steadily, it peaked among age people aged ≥ 45 years (P<0.001) | Statistical tests not well described |
| Abdella et al/1989-1990 | WHO 1985 (2) |  | Overall prevalence DM: 7.6 % | prevalence in urban area: 5.6 %; prevalence in rural area: 10.0 %; prevalence generally increased with age in both sexes in both areas; prevalence was generally greater in females (neither tested for significance)  Mean BMI was 31.8±6.3 and 28.5±5.1 in females and males respectively | Only cases sufficiently severe to merit hospital clinic attendance were identified |
| Al-Lawati & Mohammed / 1991 | WHO (1985)/ADA (1997) criteria for DM | OGGT | Prevalence of DM: 10.5 % by WHO criteria, 8.2 % by ADA criteria  Prevalence of IGT 10.5 % by WHO criteria, 5.7 % by ADA criteria | The difference in the prevalence of DM was less profound (10.5% by the WHO criteria vs. by ADA, P<0.0001) | Characteristics of population studied unclear |
| Mahfouz et al/1993 | ‘Hospital-confirmed’ (following repeat RBG > 7.8 mM) | Random blood glucose | Prevalence DM 9.7 % in males, 9.8 % in females Prevalence IGT 8.1 % in males, 12.9 % in females |  | Sampling method not clear  Nature of population registered with health centres not specified |
| El-Hazmi et al/ 1991 | WHO 1980/1985 | OGGT and IGT | The prevalence of T2DM: 4.9%  The prevalence of IGT: 0.7% | The prevalence of DM peaked in the age group≥ 30 years (P<0.001) | Limitation of the study not discussed  Steps taken to minimize bias not discussed |
| El-Hazmi et al/1991 | WHO 1980/1985 | OGGT and IGT | The overall prevalence of T2DM: 6.89%; IGT: 0.77% | 73% of female both diabetic or non-diabetic were estimated to be either obese or overweight compared to 50% of their male counterparts | Limitation of the study not discussed  Steps taken to minimize bias not discussed |
| Al-Nuaim/ 1991-1993 | WHO 1985 | OGGT and IGT | Overall prevalence DM: 12 % in urban males, 7 % rural males, 14 % urban females, 8 % rural females  Overall prevalence IGT: 10 % in urban males, 8 % in rural males, 11 % in urban females, 8 % in rural females | Age adjusted prevalence DM significantly higher in urban vs. rural population (p = 0.0001 for both male and female groups)  Prevalence of obesity (BMI > 30 kg/m2) among males and females respectively was 15% and 24% (P=0.001) | Limitation of the study not discussed  Steps taken to minimize bias not discussed |
| Al-Shammari et al/ 1993-1994 | Previously diagnosed | - | Overall prevalence DM 12.2 % |  | Methods not well described  Demographics of total population not available  Diagnosis not confirmed; sample misses more problematic cases ('referred to hospital clinic')  Sample not representative for the whole population (National Guard employees and dependants) |
| Al-Mahroos & McKelcue/ 1995-1996 | WHO 1985 | OGGT and IGT | Overall prevalence DM: 29.8 %; prevalence DM in males 40 - 49: 22.9 %; in males 50 - 59: 29.6 %; in females 50 - 59: 35.4 %; in females 60 - 69: 37.6 %  Overall prevalence IGT: 17.9 %; prevalence IGT in males 40 - 49: 16.6 %; in males 50 - 59: 15.8 %; in females 50 - 59: 19.4 %; in females 60 - 69: 22.4 %; OR for DM in women 1.27 (95 % CI 0.96 - 1.66) | 28% of subjects had BMI≥ 30 kg/m2, only 42% rated themselves as overweight. | Limitation of the study not discussed  Steps taken to minimize bias not discussed |
| Al-Mahroos and Al-Roomi/ NR | Treatment/ WHO 1985 | OGGT and IGT | overall prevalence DM 30 % |  | Method of blood sampling not reported |
| Al-Nozha et al/ 1995-2000 | ADA 1997 | OGGT and IGT | Overall prevalence DM 23.7 %; prevalence higher in males: 26.2 % (95 % CI 25.2 - 27.2) vs. females 21.5 % (95 % CI 20.6 - 22.4; p < 0.0001) (significance unclear); overall prevalence IFG 14.1 % (no gender difference) | DM more prevalent in urban (25.5 %) vs. rural (19.5 %) areas (p < 0.0001); rates of DM increased with advancing age | sample selection method not clear  Limitation of the study not discussed  Steps taken to minimize bias not discussed |
| Malik et al/ 1999-2000 | WHO 1999 | OGGT, IFG and IGT | overall prevalence DM: 21.4 % (95 % CI 20.4 - 22.4 %); prevalence in men 20.4 % (18.8 - 22.0 %); prevalence in women 22.3 % (20.9 - 23.7 %  Prevalence in UAE citizens 25 %, expats 13 - 19 %  Prevalence IFG: 4.5 % (3.7 - 5.3 %) in men, 7.2 % (6.3 - 8.1 %) in women (significantly higher in women, p < 0.01) | Roughly 22% of the sample had BMI (35-39.9)  40% had (BMI 25-29) | Selection of subjects intentionally biased towards UAE citizens |
| Al-Asi/ 2000 | Medication/ FBG > 7 mM |  | Overall prevalence of DM: 17% | Overall prevalence of overweight (BMI 25-29.9) was 48% , obesity (BMI >30) : 27% | Sample not representative for the whole population |
| Al-Moosa et al/ NR | SR/ WHO 2000 | FPG | overall prevalence DM: 11.6 % (11.8 % in males, 11.3 % in females; 17.7 % in urban population, 10.5 % in rural population); urban residence significantly associated with DM (adjusted OR) = 1.7 (95% CI 1.4–2.1), for every 5 year increase in age, 1.2 greater odds of DM (95 % CI 1.4 - 2.1) | The prevalence of obesity (BMI≥30) among sample : 21.5% | Dates of investigation unclear  Secondary data collection |
| Moussa et al/ 2000-2002 | Previous diagnosis of T2DM (made by WHO 1985 (2) and ADA 1998, 2000 criteria | FPG | Overall prevalence DM: 34.9 per 100000 (95 % CI 24.7 - 45.1); males 47.3 per 100000 (CI 28.7-65.8); females 26.3 per 100000 (CI 14.8-37.8); significantly higher prevalence T2DM in males (p = 0.05) (p = 0.013 on age-adjusted data)) and with advancing age (p = 0.026). |  | Secondary data collection |
| Baynouna et al/ 2004-2005 | ADA 2005/ medication/ SR | OGGT and IFG | Overall prevalence DM 23.3%; prevalence by age and gender: males: 5.1 % 20 - 29 years, 11.1 % 30 - 39 years, 29.5 % 40 - 49 years, 35.5 % 50 - 59 years, 55.9 % > 60 years; females: 1.7 % 20 - 29 years, 5.3 % 30 - 39 years, 26.2 % 40 - 49 years, 27.1 % 50 - 59 years, 43.3 % > 60 years; overall prevalence IFG not reported, but prevalence reached 20 % 'as early as' 20 - 24 years in males, 35 - 39 years in females | Prevalence of obesity (BMI ≥30) was greater in females than males (46.5% vs. 28.3%, P<0.01 respectively) | Statistical analysis was not described clearly  Study limitations not discussed |
| Saadi et al/ 2005-2006 | Self-reported + current oral medication/insulin, or WHO 1999 | OGGT and IGT | Overall prevalence DM: 10.2 % (9.4 % in males, 11.1 % in females); prevalence in 30 - 64 years population: 20.6 % (17.7 % in males, 22.1 % in women); prevalence IGT: 22.8 % (19.7 % in males, 24.3 % in females) |  | Potential of under- or overestimation of the reported rate of diabetes diagnosis (10.2%) based on disease reporting rate  % of subjects out of those sampled households (2455) was underwent testing was small |
| Bener et al/ 2009 | Self reported and currently taking oral medication or WHO 2006 criteria | OGGT and IGT | Overall prevalence DM: 16.7 % (15.2 % males, 18.1 % females); overall prevalence IGT: 12.5 % (12.3 % males, 12.8 % females); age significantly associated with DM (p = 0.0001, multiple logistic regression analysis); peak age DM 40 - 49 years (58 %) | Central obesity was common in 76.3%, p<0.001 |  |

**Table S2 (B): Summary of prevalence of type 2 diabetes in the GCC region**

Summary of cross-sectional studies investigating prevalence of type 2 diabetes within populations of the GCC region
