# Supplementary material for: Prevalence of Type 2 Diabetes in the States of The Co-Operation Council for the Arab States of the Gulf: A Systematic Review
Source: PLoS One. 2012 Aug 8;7(8):e40948. doi: 10.1371/journal.pone.0040948 (PMC3414510; doi:10.1371/journal.pone.0040948)
Supplement: Appendix S1 — Search Protocol. (DOCX) [file pone.0040948.s005.docx]

# Appendix S1. Review protocol.

# Prevalence of type 2 diabetes management in the states of The Co-operation Council for the Arab States of the Gulf: a systematic review

## Background

The World Economic Forum rates chronic diseases one of the ‘top 6’ Global Risks (1). They carry enormous levels of morbidity and have become major bearers of mortality. Diabetes mellitus is a chronic metabolic disorder caused by defects in insulin secretion, insulin action, or both. If ineffectively controlled, the resulting chronic hyperglycaemia is associated with numerous disabling complications, and the International Diabetes Federation (IDF) 2010 estimate suggests that diabetes mellitus accounts for 6.8 % of all-cause mortality in the 20 – 79 age group (2). 90 % of cases of diabetes mellitus are of type 2 diabetes mellitus (3), a form of diabetes characterised by insulin resistance with a relative or real insulin deficiency. Over the past 3 – 4 decades there has been a global expansion in the prevalence of type 2 diabetes, associated with population growth, ageing, urbanisation and lifestyle changes (4, 5). These trends pose a particular risk to low- and middle- income countries, where most diabetes and most deaths from diabetes occur (5), where a greater proportion of individuals affected by type 2 diabetes are of working age (< 70 years; 6), where changing demographics predict the greatest increases in prevalence, lifestyle changes anticipate relatively high increases in prevalence, interventions are likely to be fewer, and individuals generally pay a larger share of health costs.

The states of The Co-operation Council for the Arab States of the Gulf (GCC) exhibit some of the highest rates of type 2 diabetes in the world. Five of the International Diabetes Federation’s IDF ‘top 10’ countries for diabetes prevalence in 2010 and in 2030 are projected to be in this region (1). Currently, the IDF estimates suggest that in 2010 the ranking of countries by highest prevalence of diabetes starts as follows (2):1. Nauru, 2. United Arab Emirates (UAE), 3. Kingdom of Saudi Arabia (KSA), 4. Mauritius, 5. Bahrain, 6. Reunion, 7. Kuwait, 8. Oman, 9. Tongo, 10. Malaysia.

The anticipated prevalences for diabetes 2010-2030 in the Gulf countries are: United Arab Emirates (UAE) 18.7-21.4%, Kingdom of Saudi Arabia (KSA) 16.8-18.9%, Bahrain 15.4-17.3%, Kuwait 14.6-16.9% and Oman 13.4-14.9% (1). Rates in Qatar are also relatively high (15.4 % comparative prevalence). Prevalence estimates for 2030 (based only on anticipated changes in population size and demographic; 7) suggest the same will be true then. These likely underestimates (7) nevertheless anticipate prevalence in the IDF’s ‘Middle East-North Africa’ region will be 93.9 % higher in 2030 (2).

The recent and rapid socio-economic development of the GCC countries has been associated with this rising prevalence. The IDF suggests that even in the absence of further economic development (that is, based on changes in population demography alone), the number of people with diabetes in its Middle East-North Africa region will increase 94% from 2010 to 2030. Only the Sub-Saharan African region is expected to see a greater increase in the number of cases of diabetes (98%) during this period (1).

### Review question

A literature search was used to identify material relevant to the following review question:

- What is the prevalence of type 2 diabetes in populations of the GCC region?

### Inclusion criteria

**Types of studies**

Studies that used designs from the used list of acceptable methods including: observational study (cross sectional, descriptive, ecological, cohort, case-control).

**Types of participants**

Subjects residing in the GCC countries at all ages, sexes and ethnicities were included, resident and expatriate populations, urban and rural, of all socioeconomic and educational backgrounds in the GCC.

### Exclusion criteria

- Studies that used qualitative methods such as focus group and based on opinions
- Studies where population is mainly pregnant women with type 2 diabetes, or people with other types of diabetes

### Study selection

Study collection will be conducted in two stages: (1) an initial screening of the title/abstract against inclusion criteria to identify any relevant paper will be carried out by one reviewer (L.A) ; (2) screening of the full paper that identified to be relevant from the first stage, and it will be carried out by 2 reviewers (L.A and A.M). If disagreement regarding any study eligibility appears, it would be resolved through discussion and asking for the opinion of the third reviewer (A. M).

No limitations on publication type, publication status, study design or language of publication will be imposed.

### Data extraction/quality assessment

The data captured for each study will include data relating to, (1) methods (study design, recruitment, measurement tools, analysis); (2) participant characteristics (3) setting, and (4) outcomes (those observed, their definitions, results of analysis, length of follow-up). Study quality was assessed using a checklist adapted from the Centre for Reviews and Dissemination guidelines (8). Data extraction will be performed, in duplication, by two reviewers (L.A, A.M).

### Data synthesis

.

Synthesis will include summarising the results of the data extraction process, considering the strength of evidence relating to each of our questions, and examination of results inconsistent with our formed proposals.

### References

1. Global Risks 2010: A Global Risk Network Report, World Economic Forum, January 2010, © 2010 World Economic Forum
2. World Health Organization. *Preventing chronic diseases: a vital investment.* Geneva: World Health Organization; 2005
3. International Diabetes Federation (2009) IDF Diabetes Atlas, 4^th^ ed. (c) International Diabetes Federation, 2009.
4. World Health Organisation (2009) Fact sheet N°312 Diabetes
5. Amos, A., McCarty, D. & Zimmet, P. The rising global burden of diabetes and its complications: estimates and projections to the year 2010. *Diabetic Med.* 14, S1-S85 (1997).
6. King, H., Aubert, R. & Herman, W. Global burden of diabetes, 1995-2025. Prevalence, numerical estimates and projections. *Diabetes Care* 21, 1414-1431 (1998).
7. Centre for Reviews and Dissemination (2009) Systematic reviews: CRD's guidance for undertaking reviews in health care [Internet]. York: University of York; 2009 [accessed July 2010]. Available from: <http://www.york.ac.uk/inst/crd/systematic_reviews_book.htm>
