# Supplementary material for: Prevalence of Type 2 Diabetes in the States of The Co-Operation Council for the Arab States of the Gulf: A Systematic Review
Source: PLoS One. 2012 Aug 8;7(8):e40948. doi: 10.1371/journal.pone.0040948 (PMC3414510; doi:10.1371/journal.pone.0040948)
Supplement: Appendix S2 — Research questions using PICO. (DOCX) [file pone.0040948.s006.docx]

**Appendix S2. Research questions using PICO.**

- **Research question:** What is the prevalence of type 2 diabetes in the populations of the GCC region?

AND

| **Patient/Population** | **Outcomes** |
| --- | --- |
| People living in the GCC | Prevalence of: type 2 diabetes |
| **GCC:** Qatar, United Arab Emirates UAE, Kingdom of Saudi Arabia KSA, Kingdom of Bahrain, Sultanate of Oman, Kuwait | **Prevalence:** statistics, epidemiology  **Type 2 diabetes mellitus:** Diabetes mellitus, Non-insulin dependent diabetes mellitus, T 2 DM, impaired glucose tolerance, MODY or NIDDM, diabetes insipidus |
