# Supplementary material for: Prevalence of Type 2 Diabetes in the States of The Co-Operation Council for the Arab States of the Gulf: A Systematic Review
Source: PLoS One. 2012 Aug 8;7(8):e40948. doi: 10.1371/journal.pone.0040948 (PMC3414510; doi:10.1371/journal.pone.0040948)
Supplement: Appendix S3 — Search strategy. (DOCX) [file pone.0040948.s007.docx]

**Appendix S3. search strategy.**

**Prevalence of type 2 diabetes in the states of The Co-operation Council for the Arab States of the Gulf: a systematic review**

**Search strategy to identify studies from electronic databases**

The followed steps for the search strategy included: (1) formatting a well defined review question to maintain the transparency for the review process; (2) revising the review question using PICOS elements (Population, Intervention, Comparators, Outcomes, Study design) (appendix 2); (3) defining the inc/exclusion criteria for the study; (4) producing a list of synonyms abbreviations and spelling variants; (5) combining the PICOS elements using Boolean logic (AND, OR); (6) devising a search strategy using both indexing terms and free text; (6) reviewing the search strategy; (7) pilot the search strategy on one database EMBASE; (8) review the search strategy with another colleague (w. I); (9) repeat the search strategy and finalize it.

**Describing electronic database searches**

**Describing electronic database searches**

The Medline and Embase were searched separately on 15/07/2009 and the search was repeated on 03/07/2010 (via Dialog and Ovid, respectively; 1950 to July week 1, and 1947 to 2010 July) using the following search strategy:

**Type 2 diabetes**

| 1. exp diabetes mellitus, non-insulin-dependent/ |
| --- |
| 1. exp insulin resistance/ |
| 1. impaired glucose toleranc$.tw. |
| 1. glucose intoleranc$.tw. |
| 1. insulin$ resistanc$.tw. 2. (MODY or NIDDM).tw. 3. ((typ$ 2 or typ$ II) adj diabet$).tw. |
|  |
|  |
| 1. ((keto?resist$ or non?keto$) adj diabet$).tw. |
| 1. ((adult$ or matur$ or late or slow or stabl$) adj diabet$).tw. |
| 1. (insulin$ defic$ adj relativ$).tw. |
| 1. pluri?metabolic$ syndrom$.tw. |
| 1. 1 or 2 or 3 or 4 or 5 or 6 or 7 or 8 or 9 or 10 or 11 |
| 1. exp diabetes insipidus/ |
| 1. diabet$ insipidus.tw. 2. 13 or 14 3. 12 or 15 4. Exp prevalence/ 5. Exp epidemiology/ 6. Stat$.tw. 7. 17 or 18 or 19   **The states of The Co-operation Council for the Arab States of the Gulf (GCC)**   1. ((Saudi or emirates or Kuwait or Oman or Bahrain or Qatar) adj5 (middle east* or Arab*)).mp. [mp=title, original title, abstract, name of substance word, subject heading word, unique identifier]   **Prevalence of type 2 diabetes in the GCC**   1. 21 and 20 2. 16 and 20 |

**Describing journal hand searches**

1. International Journal of Diabetes and Metabolism searched for the period 1993 to 2009
2. Saudi Medical Journal for the period 2000 to 2010

**Describing the methods used to search relevant internet sources**

1. The International Diabetes Federation (2009) IDF **(**<http://www.diabetesatlas.org/>) was searched using the on-site search engine. The section of the website labelled Diabetes Atlas 4^th^ ed. was searched in detail.
2. The World Health Organisation (2009) WHO **(**[**http://www.WHO.int/mediacentre/factsheets/fs312/en/**](http://www.WHO.int/mediacentre/factsheets/fs312/en/)**)** was searched using on-site search engine. The section of the website labelled Fact sheet N 312 Diabetes was searched in detail.

**Describing other searches included**

1. The reference lists of included studies in the review were scanned for relevant studies.
